# Prolonged response to PIK3CA inhibition in an advanced hormone-positive breast cancer patient with 3 mutations in the alpha subunit of PI-3 kinase

**DOI:** 10.1093/oncolo/oyaf126

**Published:** 2025-06-19

**Authors:** Rotem Merose, Raya Leibowitz

**Affiliations:** Oncology Institute, Shamir Medical Center, Be’er Yaakov 703000, Israel; Oncology Institute, Shamir Medical Center, Be’er Yaakov 703000, Israel; Oncology Department, Faculty of Health and Medical Sciences, Tel Aviv University , Tel Aviv 69978, Israel

**Keywords:** breast cancer, PIK3CA, alpelisib, mutation

## Abstract

Mutations in the oncogene PIK3CA are implicated in many types of solid tumors and are prevalent in estrogen-receptor (ER)-positive Her2-negative (ER+Her2−) breast cancer (BC). In recent years, a combination of a drug-modulating ER and a PIK3CA inhibitor was approved for PIK3CA-mutant BC. We present a case of a 69-year-old otherwise healthy woman who was diagnosed with early ER+Her2− BC at the age of 47 and was treated with lumpectomy, adjuvant chemotherapy, radiation, and adjuvant tamoxifen. Five years after the termination of chemotherapy, she was diagnosed with bone metastasis and received several lines of endocrine therapy and subsequent capecitabine for 17 years altogether. Upon disease progression in the liver and lung, a “liquid biopsy” from the blood detected 3 missense mutations in PIK3CA, one of which was known to be deleterious (H1047R). The patient started a combination of Fulvestrant and Alpelisib with a deep and prolonged clinical and radiological response for 3 years. The occurrence of multiple mutations in PIK3CA was documented in 12-15% of BCs. A retrospective analysis showed that ER+Her2− BC patients with multiple PIK3CA mutations had a significantly higher response rate to PIK3CA inhibitor. It is impossible to determine whether our patient’s extremely prolonged progression-free survival on Alpelisib-Fulvestrant was a result of the multiplicity of mutations in PIK3CA, the existence of the known deleterious mutation H1047R, or other clinical/biological factors. Our case highlights the potential importance of the number of PIK3CA mutations observed in a pathological sample or liquid biopsy, despite it being still unknown how to incorporate the multiplicity or singularity of PIK3CA mutations into clinical decision-making.

**Key points**

- Mutations in the oncogene PIK3CA are implicated in many types of solid tumors and are very prevalent in estrogen-receptor (ER)-positive Her2-negative (ER+Her2−) advanced breast cancer (BC). In recent years, a combination of a selective ER degrader and a PIK3CA inhibitor was approved for this indication.
- We present a case of a patient with ER+Her2− advanced BC, previously treated with several lines of hormonal therapy and capecitabine, who was found to have 3 mutations in PIK3CA by liquid biopsy. The patient had a deep and prolonged response to Fulvestrant and Alpelisib for 3 years.
- Our case is in keeping with recent evidence that mutation multiplicity in PIK3CA is not rare and that the number of observed mutations may be associated with clinical outcome. Our case exemplifies the importance of this potentially overlooked parameter.

## Patient story

A 69-year-old otherwise healthy woman was diagnosed with early estrogen-receptor (ER) positive, Her2-negative (ER+Her2−) breast cancer (BC) at the age of 47 (in 1998) and was treated at a different hospital with lumpectomy, adjuvant chemotherapy, radiation, and adjuvant tamoxifen. Five years following completion of chemotherapy, she was diagnosed with bone metastasis and received several lines of endocrine therapy (the exact details are unknown), after which she received capecitabine for 6 years, up until 2016. Subsequently, the disease progressed to lung and liver. Endocrine therapy was then resumed; she received single-agent letrozole, followed by single-agent Fulvestrant, and in January 2018 started Exemestane and Palbociclib for about a year, after which capecitabine was resumed for a few more years. At around that time, she started treatment at our center.

In January 2021, her scans revealed initial signs of disease progression in the liver, and by March 2021 there was clear progression in the liver, accompanied by exacerbation of her chronic cough (attributed to her known lung metastasis) and the appearance of submandibular lymphadenopathy.

## Molecular tumor board

### Genotyping results and interpretation of the molecular results

As the patient did not wish to undergo an invasive procedure, she opted to undergo a “liquid biopsy” using a commercially available kit (Guardant360) in February 2021, detecting the following alterations: mutation H1047R in PIK3CA in 0.6% of the cfDNA, mutation R108C in PIK3CA in 0.3% of the cfDNA, mutation C804R in PIK3CA in 0.2% of the cfDNA, mutations S241C and N239D in TP53 in 0.4% and 0.3% of cfDNA, respectively, and mutation G2695C in ATM in 0.2% of cfDNA. No mutations were found in BRCA1/2, ESR1, and ERBB2. The micro-satellite instability was low.

### Functional and clinical significance of the specific mutation in the particular cancer

The use of liquid biopsies has increased in recent years across all the spectrum of solid malignancies, including BC, and is advised at times of disease progression (although not yet considered “standard of care,” partly due to reimbursement issues). A recent characterization of the Landscape of circulating tumor DNA in metastatic BC revealed that 88% of patients had at least one ctDNA alteration detected, the most common of which was PIK3CA.^1^

The PIK3CA-AKT-mTOR pathway has long been implicated in BC, and dozens of clinical trials have aimed, for more than 2 decades, to target several of its components in this malignancy. Mutations in PIK3CA occur in many types of solid cancers, and specifically in 25%-40% of ER+ BCs.^2^ These observations have led to the pharmacological development of PIK3CA inhibitors. The pivotal SOLAR-1 trial demonstrated that the addition of PIK3CA inhibitor Alpelisib to the selective ER degrader Fulvestrant significantly prolonged progression-free survival (PFS) in ER+Her2− BC and established this biological-hormonal combination in this subset of biomarker-positive patients.^3^ Later, the combination of the AKT-inhibitor capivasertib and fulvestrant was shown to improve progression-free survival in ER+her2− metastatic BC relative to fulvestrant alone in patients with AKT pathway-altered (PIK3CA, AKT1, or PTEN) tumors.^4^

Our patient was found to have 3 mutations in her circulating free DNA.^5^ The PIK3CA mutation H1047R is a prevalent missense mutation in the kinase domain of PIK3CA that increased PIK3CA oncogenicity.^6^ Indeed, its occurrence was associated with response to PIK3CA/AKT/mTOR inhibitors.^7^ The PIK3CA mutation R108C lies within the p85 domain of the Pik3ca protein and has transformative abilities in vitro.^8^ The PIK3CA mutation C604R lies within the PIK helical domain of the Pik3ca protein. C604R results in increased cell proliferation, migration, and Akt phosphorylation compared to wild-type Pik3ca and increased transformation ability in several different cell lines in culture,^8,9^ and thus can be defined as a “gain of function” mutation (Table 1).

**Table 1.** Mutations in PIK3CA found in the liquid biopsy.

Mutation	Prevalence (%of cfDNA)	Type	Location	Pathogenicity	Reference
H1047R	0.6	Missense	Kinase domain	Deleterious	^5^
R108C	0.3	Missense		Unknown	^10^
C604R	0.2	Missense	Helical domain	Unknown	^11^

### Potential strategies to target the pathway and implications for clinical practice

In an elegant work, Vasan et al. demonstrated the existence of multiple mutations in PIK3CA in 12-15% of BC; in most cases, the mutations existed in cis (ie, on the same allele) and resulted in increased PIK3CA activity, enhanced downstream signaling and increased tumor growth.^12^ Moreover, retrospective molecular analysis of data from the SANDPIPER trial, assessing the PIK3CA inhibitor Taselisib in mutant ER+Her2− advanced BC patients showed that patients with multiple PIK3CA mutations had a significantly higher response rate to Taselisib/Fulvestrant than to placebo/Fulvestrant (30.2% vs 8.7%, respectively).^13^ Clearly, liquid biopsy results cannot determine whether the 3 mutations found in the cfDNA of our patient stemmed from one or both alleles. It is also unknown whether there is a “dose-response” relationship between the number of the mutations detected and clinically meaningful outcome such as response, PFS or overall survival (OS).

## Patient update

In March 2021, the patient was started on Fulvestrant (given as re-introduction as she received it previously) and Alpelisib at an initial dose of 300 mg, which was quickly reduced to 250 mg due to the appearance of a drug rash. Within 10 days, a clear improvement in the cough and a clear decrease in the size of the palpable submandibular lymph node was observed. Her first scan, 4 months after initiation of treatment, demonstrated a deep partial response in the liver, lung, lymph nodes, and bones. After a few months of treatment, hyperglycemia appeared, which was well-managed on metformin. She also complained of slight asthenia and lack of appetite.

The patient received the drug combination for 3 years; approximately 18 months into the treatment course, an oligoprogression occurred within a single liver metastasis, that was irradiated stereotactically with a good response, while continuing the same antineoplastic systemic treatment.

In March 2024, progressive disease in the liver, lung, and lymph nodes was observed, the treatment was stopped and the patient started paclitaxel, with good clinical response.

It is impossible to determine whether the deep and prolonged response to Alpelisib/Fulvestrant stemmed from the existence of the known deleterious mutation H1047R, the occurrence of 3 concurrent mutations on PIK3CA, other biological/clinical factors, or a combination of all of these. Of note, at a median follow-up of 20 months, the median PFS on the experimental arm in the SOLAR-1 trial was 11 months, with a 95% confidence interval of 7.5-14.5 months. Our patient’s PFS was more than 3 times the median of the trial, with her previously progressing on single-agent Fulvestrant.

Our case highlights the potential importance of the number of PIK3CA mutations observed in a given pathological sample of liquid biopsy, yet many questions, remain. It is currently unknown how to incorporate the multiplicity or singularity of PIK3CA into clinical decision-making, and specifically whether the mutation number should affect sequencing of hormonal lines of therapy, or even if it should aid in choosing additional lines of hormonal therapy over chemotherapy.

Our case also exemplifies the growing need of the medical oncologist to remain “up-to-date” with the ever-increasing body of literature on molecularly targeted therapies.
